# Identification of gene expression logical invariants in *Arabidopsis*

**DOI:** 10.1002/pld3.123

**Published:** 2019-03-20

**Authors:** Sonalisa Pandey, Debashis Sahoo

**Affiliations:** ^1^ University of California San Diego San Diego California

**Keywords:** bioinformatics, Boolean analysis, microarray, systems biology

## Abstract

Numerous gene expression datasets from diverse tissue samples from the plant variety *Arabidopsis thaliana* have been already deposited in the public domain. There have been several attempts to do large scale meta‐analyses of all of these datasets. Most of these analyses summarize pairwise gene expression relationships using correlation, or identify differentially expressed genes in two conditions. We propose here a new large scale meta‐analysis of the publicly available *Arabidopsis* datasets to identify Boolean logical relationships between genes. Boolean logic is a branch of mathematics that deals with two possible values. In the context of gene expression datasets we use qualitative high and low expression values. A strong logical relationship between genes emerges if at least one of the quadrants is sparsely populated. We pointed out serious issues in the data normalization steps widely accepted and published recently in this context. We put together a web resource where gene expression relationships can be explored online which helps visualize the logical relationships between genes. We believe that this website will be useful in identifying important genes in different biological context. The web link is http://hegemon.ucsd.edu/plant/.

## 1 INTRODUCTION

A large amount of microarray and RNASeq datasets has been continually deposited in public databases such as GEO (Gene Expression Omnibus) (Barrett et al., 2005, 2013; Edgar, Domrachev, & Lash, 2002) and ArrayExpress (Brazma et al., 2003; Rocca‐Serra et al., 2003). It is challenging to put together all of these data from different labs or different studies in order to facilitate comparisons across labs and diversity of tissue types; however, there have been several attempts of massive large scale data analysis that provides new hypothesis and insight into biological processes. NASCArrays is one of the first few that started this revolution in the plant community (Craigon et al., 2004). Following this, several studies have put together large databases (Ball et al., 2005; He et al., 2016; Lukk et al., 2010; Schmid, Palmer, Kohane, & Berger, 2012; Zimmermann, Hirsch‐Hoffmann, Hennig, & Gruissem, 2004), and web resources to investigate gene‐gene relationships online (Katari et al., 2010; Manfield et al., 2006; Mutwil, Obro, Willats, & Persson, 2008; Obayashi & Yano, 2014; Srinivasasainagendra, Page, Mehta, Coulibaly, & Loraine, 2008; Toufighi, Brady, Austin, Ly, & Provart, 2005). The GeneMANIA App in Cytoscape also incorporates co‐expression analysis of transcriptomic datasets (Montojo et al., 2010). The Arabidopsis Information Resource (TAIR) provides an integrative platform where many different datatypes, including gene expression, can be effectively analyzed (Rhee et al., 2003).

Despite these largescale efforts, none of the above resources provide interfaces to analyze Boolean logical relationships between genes. Boolean logic is mathematics of two possible values. In the context of gene expression data, one might ask what other genes are highly expressed if the expression of gene A is high. Boolean logical gene‐gene relationship is mathematically the simplest form of relationship between genes. We have published earlier how Boolean relationship can be explored in large microarray datasets (Sahoo, 2012; Sahoo, Dill, Gentles, Tibshirani, & Plevritis, 2008). Boolean relationship is identified by searching for at least one sparsely populated quadrant out of four possible quadrants by the BooleanNet algorithm (Sahoo et al., 2008). According to this BooleanNet algorithm, there are six potential Boolean implications of gene relationships: two symmetric Boolean implications (Equivalent and Opposite) and four asymmetric Boolean implications (Sahoo et al., 2008). Two genes are considered Boolean equivalent if they are positively correlated with only high‐high and low‐low gene expression values. Two genes are considered Boolean opposite if they are negatively correlated with only high‐low and low‐high gene expression values. Asymmetric Boolean implications result when there is only one sparsely populated quadrant.

In this paper, we put together a web resource for the plant community to explore Boolean logical gene‐gene relationships. In addition, we describe special types of relationships called logical invariants in detail. An invariant is a formula that evaluates to true in a universe of sample types. A universe consists of a coherent set of samples from a particular tissue. The plant universe consists of all plant tissue types. All samples from roots can form a universe: the root‐universe. Similarly, we can have a shoot, leaf, and flower universe. A logical invariant is associated with a particular universe where it evaluates to true. A Boolean logical gene‐gene relationship will be called a logical invariant if all possible samples from that universe follow the same logical rule. Therefore, it is hard to call any relationship logical invariant because we do not have access to all possible samples. However, we could hypothesize a Boolean relationship logical invariant if the relationship looks strong. In this paper, we will identify several candidate logical invariants in all plant tissues as well as specific tissue types.

## 2 METHODS

### 2.1 Data collection and annotation

Publically available microarray databases in *Arabidopsis thaliana* ATH1 (number of microarray samples in 2014 *n* = 4,306, GPL198) Affymetrix platform were downloaded from the National Center for Biotechnology Information (NCBI) GEO website (Barrett et al., 2005, 2013; Edgar et al., 2002). Gene expression summarization was performed by normalizing each Affymetrix platform by Robust Multi‐Array Average (RMA) (Irizarry et al., 2003). We downloaded the latest dataset where tissue type, growth conditions, and developmental stage were manually curated for each sample (GSE69995, *n* = 6,057) (He et al., 2016). In addition to these large datasets which are mostly bulk tissue datasets, we put together a couple of datasets where specific cell types were purified using Fluorescence‐Activated Cell Sorting (FACS) method. The Yadav‐2014 dataset that provides a high resolution map of the shoot apical meristem (SAM) cell types of central zone (CZ), peripheral zone (PZ), and rib meristem (RM) (GSE28109, GSE13596)(Yadav, Girke, Pasala, Xie, & Reddy, 2009; Yadav, Tavakkoli, Xie, Girke, & Reddy, 2014) was prepared. Similarly, the Benfey dataset that provides a high‐resolution spatiotemporal map of the root (GSE15876, GSE16468, GSE16469, GSE21582, GSE30166, GSE35580, GSE5749, GSE61408, GSE64253, GSE7641, and GSE8934) (Bargmann et al., 2013; Birnbaum et al., 2003; Brady et al., 2007; Carlsbecker et al., 2010; Dinneny et al., 2008; Efroni, Ip, Nawy, Mello, & Birnbaum, 2015; Iyer‐Pascuzzi et al., 2011; Lee et al., 2006; Long et al., 2010; Nawy et al., 2005; Sozzani et al., 2010) was prepared. Both the Yadav‐2014 and Benfey dataset use specific reporter lines and purify specific cell types and profile them using the *Arabidopsis thaliana* ATH1 Affymetrix platform. We have also collected multiple RNASeq datasets (*n* = 747) using previously published tool by Zhuo, Emerson, Chang, and Di (2016) StablyExpressedGenes. We prepared these RNASeq datasets by computing TPM (Li & Dewey, 2011; Pachter, 2011) values using a custom perl script. We used log2(TPM) if TPM > 1 and (TPM − 1) if TPM < 1 as the final gene expression value.

### 2.2 Duplicate CEL files identification

Following the 330 experiments from the previously published dataset GSE69995, 6,535 CEL files were downloaded from GEO. 6,057 CEL files were used to build the published dataset GSE69995. Quality control steps from the “simplyaffy” and “affyPLM” data packages (Bolstad, Irizarry, Astrand, & Speed, 2003) were used before to identify these 6,057 CEL files that exclude 478 files (6,535–6,057) from our list. We computed a MD5 hash of each file and compared them to check if there were duplicate entries under a different file name. If two files were identical, their MD5 hash was matched even if the file names were different. In 6,535 CEL files, we found a total of 87 duplicated entries (Supporting Information Table S1) and 85 duplicated entries were present in the published dataset GSE69995. We created a new dataset based on this after removing all 85 duplicates from GSE69995, with a total of 5,972 files (6,057 − 85 = 5,972). Our dataset is available at GEO using the accession no GSE118579.

### 2.3 Boolean analysis of datasets

The expression values of each gene were ordered from low to high and a rising step function was computed to define a threshold by StepMiner algorithm in the individual dataset (Sahoo, Dill, Tibshirani, & Plevritis, 2007). If the assigned threshold for a gene was t, then expression levels above *t* + 0.5 were classified as “high”, and the expression levels below *t *− 0.5 were classified as “low”. Expression levels between *t* − 0.5 and *t* + 0.5 were classified as “intermediate”. A previously published BooleanNet algorithm was performed to determine Boolean Implication relationships between genes. Briefly, the BooleanNet algorithm searches for at least one sparsely populated quadrant in a scatterplot between two genes. The “intermediate” expression values were ignored by the BooleanNet algorithm. There were six possible scenarios: one of the four quadrants was sparse (four asymmetric Boolean implications) and two diagonally opposite quadrants were sparse (Equivalent and Opposite Boolean implications). We used the same thresholds as in our previously published algorithm: statistic > 3 and error‐rate < 0.1.

### 2.4 Web‐based visualization

Boolean implication relationships were visualized using two dimensional scatterplots between two genes. The scatterplot shows the normalized expression values of two genes along with the thresholds that separate the high and low values. Sparsely populated quadrants can be immediately spotted by visual inspection. Each individual point in the scatterplot belongs to a particular sample that can be traced to its original source at GEO with a GEO accession number. The samples in the plot can be selected using a mouse by dragging a rectangle in the plot. A group was created with the number of the sample shown on the right side of the scatterplot. The interface lets the user supply two genes at the top in two different textboxes. The textbox can be used to input a set of genes separated by whitespace. When the user clicks on “getPlots”, all possible pairs of probesets derived from the two sets of genes are plotted.

### 2.5 Comparison of Boolean networks between GSE69995 and our (Pandey 2018) dataset

A direct head‐to‐head comparison with a large dataset identified by GEO accession number GSE69995 was performed to check if data processing steps influence the discovery of logical relationships (He et al., 2016). We re‐processed the same dataset using our Boolean analysis pipeline. We used RMA to normalize the dataset while the GSE69995 dataset was normalized using MAS 5.0(Hubbell, Liu, & Mei, 2002). We matched the probeset IDs of the two datasets using the Affymetrix annotation for GPL198 which is the GEO accession number of the *Arabidopsis thaliana* ATH1 Affymetrix platform. We computed the full Boolean implication network in both datasets. For each probeset ID A we discovered six different possible Boolean implication relationships: A low ⇒ X high (lohi), A low ⇒ X low (lolo), A high ⇒ X high (hihi), A high ⇒ X low (hilo), A equivalent X (eqv), and A opposite X (opo). We plotted the number of relationships identified in both datasets in a scatter plot with a log‐log scale to compare the approaches.

## 3 RESULTS

### 3.1 Identification of duplicate entries in previous datasets

To gain more insight into gene function in plants, it is important to study tissue‐specific gene activity under a variety of conditions. However, the analysis of public expression data by the plant research community is hampered by the lack of consistent sample annotation. Searching keywords in the metadata fields for each expression sample in the GEO, such as “Characteristics,” “Description”, and “Source name” is not reliable because of inconsistent annotations. We discovered a largescale meta‐analysis of previously published datasets in GEO (GSE69995) (He et al., 2016). This dataset consists of a carefully annotated description of each sample. Therefore, it was relevant for our study to investigate logical relationships between genes. It is important to understand that accurate annotation is key to success. It is also important to remove any technical biases which may hamper further interpretation from the dataset. For example, if a sample is duplicated several times in a particular dataset, it may lead to unintended consequences in the analysis and interpretation. We discovered 85 duplicated entries in this dataset. While these duplicates may not be highly significant relative to the scale of the dataset, they should be removed before any meta‐analysis is performed. Supporting Information Table S1 lists all the duplicated entries in this dataset. The plant community should be aware of such samples in the dataset.

### 3.2 Identification of six possible types of logical invariants

**Figure 1:**
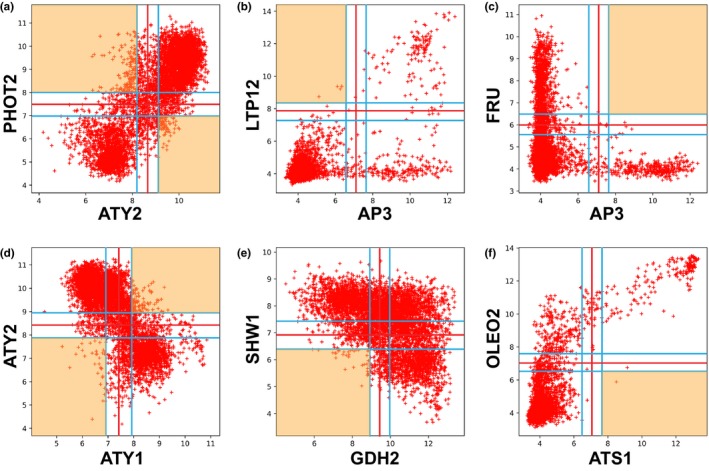
Six possible logical gene‐gene relationships. Every point in the plot is a microarray experiment performed in the ATH1 Affymetrix platform. The *x*‐ and *y*‐axes represent log2 normalized gene expression values. Sparsely populated quadrants are highlighted with orange. (a, d) Symmetric relationships. (b, c, e, f) Asymmetric relationships. (a) ATY2 equivalent PHOT2. (b) AP3 low ⇒ LTP12 low. (c) AP3 high ⇒ FRU low. (d) ATY1 opposite ATY2. (e) GDH2 low ⇒ SHW1 high. (f) ATS1 high ⇒ OLEO2 high

A full Boolean implication network was created using the new dataset. The analysis identified six possible types of Boolean implication relationships. Figure 1 shows an example of each that might be associated with some known gene functions in plants. For example, ARABIDOPSIS THIOREDOXIN Y2 (ATY2) and PHOTOTROPIN 2 (PHOT2) have a logical equivalent relationship as the top‐left and bottom‐right quadrants are sparse (Figure 1a). PHOT2 is a membrane‐bound protein serine/threonine kinase that functions as a blue light photoreceptor (Sakai et al., 2001). ATY2 is mainly expressed in leaves and induced by light (Collin et al., 2004). ARABIDOPSIS THIOREDOXIN Y1 (ATY1) and ATY2 have clear opposite relationship (Figure 1d). ATY1 is mainly expressed in non‐photosynthetic organs including seeds (Collin et al., 2004). When the APETALA 3 (AP3) expression level is low, the LIPID TRANSFER PROTEIN 12 (LTP12) expression level is also low (Figure 1b, AP3 low ⇒ LTP12 low, LTP12 high ⇒ AP3 high). AP3 is mainly expressed in flower petal and stamen (Bowman, Smyth, & Meyerowitz, 1989), while LTP12 is expressed specifically in anther and pollen (Li et al., 2014). This is consistent with the logical relationship demonstrated by LTP12 expression in a subset of tissues in flower, anther, pollen, and stamen. FER‐LIKE REGULATOR OF IRON UPTAKE (FRU) is mainly expressed in the root (Bauer et al., 2004), therefore it is consistent with the logical relationship of AP3 high ⇒ FRU low (Figure 1c). Figure 1e shows the relationship between GLUTAMATE DEHYDROGENASE 2 (GDH2) and SHORT HYPOCOTYL IN WHITE LIGHT1 (SHW1): GDH2 low ⇒ SHW1 high. The three different quadrants in the scatterplot between GDH2 and SHW1 are populated with three different tissues: roots (bottom right, GDH2 high SHW1 low), seedlings (top right, GDH2 high SHW1 high), and leaves (top left, GDH2 low SHW1 high). When ARABIDOPSIS THALIANA SEED GENE 1 (ATS1) expression is high, OLEOSIN 2 (OLEO2) expression is also high (Figure 1f, ATS1 high ⇒ OLEO2 high, OLEO2 low ⇒ ATS1 low). Both OLEO2 and ATS1 are mainly expressed in seeds (Kim, Hsieh, Ratnayake, & Huang, 2002; Nuccio & Thomas, 1999). All of the six possible relationships described above are strong in all data points. In other words, almost all of the data points follow the Boolean formula. Therefore, they are candidates for logical invariants in plants.

### 3.3 Comparison of Boolean network with previous dataset

**Figure 2:**
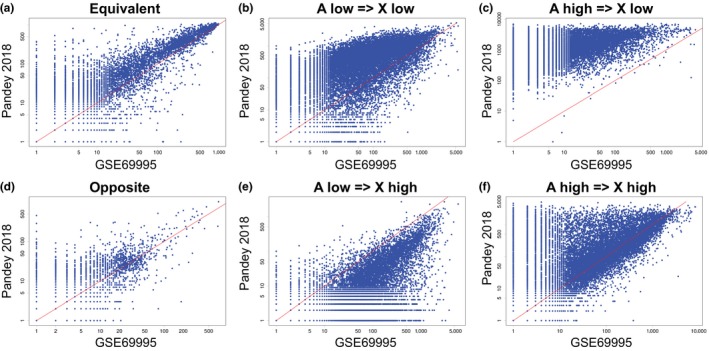
Comparison of Boolean network between GSE69995 and Pandey 2018 dataset. Every point in the plot is a probeset ID in the ATH1 Affymetrix platform. The *x*‐ and *y*‐axes represent log2 count of the respective logical relationships. *x* = *y* is plotted with a red line. (a, d) Symmetric relationships. (b, c, e, f) Asymmetric relationships. (a) A equivalent X. (b) A low ⇒ X low. (c) A high ⇒ X low. (d) A opposite X. (e) A low ⇒ X high. (f) A high ⇒ X high. Our approach discovered more significant logical relationships than the previously published dataset in all other cases except A low ⇒ X high. Gene A and Gene X are candidate probeset IDs in each dataset

A detailed comparison was performed between our dataset (Pandey 2018) and the previously published He‐2016 dataset GSE69995 to check if discovery of logical relationships was sensitive to the data processing steps. He et al. (2016) used MAS 5.0 normalization, while we used RMA. Figure 2 shows the number of relationships for each probesets in both dataset using scatterplots. *X*‐axes represent GSE69995, *y*‐axes represent our approach, and the different scatterplots correspond to the six possible logical relationships. As can be seen in the figure, our approach identified significantly more logical relationships in all other comparisons except A low ⇒ X high (Figure 2e). The *p*‐value was less than 0.001 for equivalent, opposite, lolo, hihi, and hilo. Boolean approach discovered more A low ⇒ X high in GSE69995 compared to our dataset. We conclude that the Boolean approach is best suited for data processing steps using RMA. Below we describe a few reasons why our algorithm did not find many statistically significant relationships in GSE69995.

**Figure 3:**
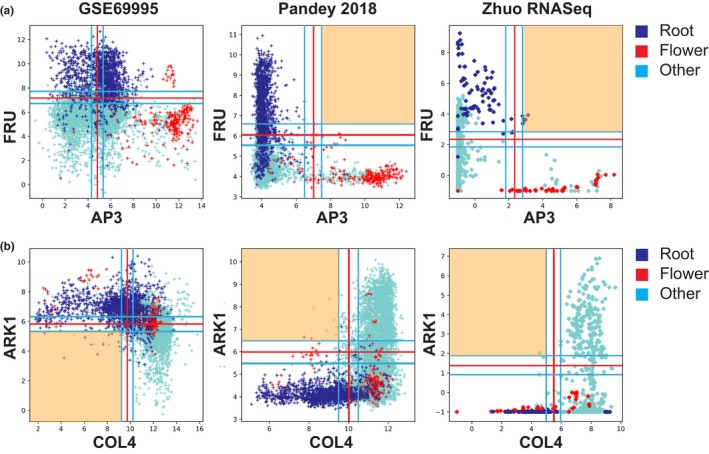
Data normalization and Boolean implication. In GSE69995 and Pandey 2018 datasets every point in the plot is a microarray experiment in the ATH1 Affymetrix platform. In Zhuo RNASeq datasets every point is an RNASeq experiment in a Arabidopsis tissue sample. The *x*‐ and *y*‐axes represent log2 normalized expression values. Root and flower tissue samples are highlighted with dark blue and red, respectively. (a) GSE69995: no relationship, some flower samples may express high levels of FRU; Pandey 2018, Zhuo RNASeq: AP3 high ⇒ FRU low, FRU is expressed in roots and AP3 is expressed in flower samples. (b) GSE69995: COL4 low ⇒ ARK1 high; ARK1 is expressed in root samples; Pandey 2018, Zhuo RNASeq: COL4 low ⇒ ARK1 low, ARK1 is not expressed in root samples

To get a deeper insight into the structure of the Boolean network, we show four scatterplots that demonstrate the discrepancy between datasets. Figure 3a shows a scatterplot between AP3 and FRU in three datasets including GSE69995, Pandey 2018, and Zhuo RNASeq. There is no significant logical relationship in the GSE69995 dataset, whereas our dataset and the RNASeq dataset show very clear AP3 high ⇒ FRU low relationship. In the scatterplots, root and flower tissue samples are highlighted in dark blue and red, respectively. The GSE69995 dataset shows that FRU is highly expressed in root samples, AP3 is high in flower samples, but some of the flower samples may have high levels of FRU, and many root samples may have high levels of AP3. However, both our dataset and the RNASeq dataset shows that all of the flower samples have low to intermediate levels of FRU, and all root samples have low levels of AP3. Our data suggest that FRU and AP3 expression levels are clearly mutually exclusive which is consistent with the literature (Jakoby, Wang, Reidt, Weisshaar, & Bauer, 2004; Kramer & Irish, 1999). These scatterplots also highlight that there may be discrepancy in identifying threshold in many genes as the threshold of AP3 is remarkably different in both datasets. Figure 2c shows a clear increase in the frequency of A high ⇒ X low in our dataset compared to the GSE69995 dataset. We hypothesize that this increase is due to the discrepancy in identifying threshold in two datasets.

Figure 2e suggested that the frequency of A low ⇒ X high is higher in the GSE69995 dataset compared to our dataset and the Zhuo RNASeq dataset. We hypothesize that there may be technical issues associated with the discovery of A low ⇒ X high in the GSE69995 dataset. Figure 3b shows a scatterplot between CONSTANS‐LIKE 4 (COL4) and ARABIDOPSIS THALIANA RECEPTOR KINASE 1 (ARK1). In the GSE69995 dataset the relationship is COL4 low ⇒ ARK1 high. It also suggests that ARK1 expression levels are higher in root samples compared to other samples. In contrast, both our dataset and the RNASeq dataset suggest that the relationship is COL4 low ⇒ ARK1 low, and the expression levels of ARK1 in root samples are low. Literature is consistent with our observation, since Northern blot analyses prepared from various tissues including floral bud, leaf, root and stem only detected ARK1 expression in leaf and floral bud (Tobias, Howlett, & Nasrallah, 1992). In the TAIR database, ARK1 is annotated as “not expressed” in root. We observed that A low ⇒ X high is usually rare in human and mouse datasets. The overwhelmingly high frequency of A low ⇒ X high in GSE69995 may be due to a technical bias.

### 3.4 Web resource for easy exploration

We provide a web interface where the gene expression data can be explored using two dimensional scatterplots. Using this interface, the user can start with two well defined gene names and query the database to plot the normalized expression values in a scatterplot. Each individual data point in the scatterplot is linked to the GSE accession number. The website provides a link to the GEO website where details of the experiment are found. The website has several features to explore Boolean logical relationships between genes. Using a mouse, the user can select a set of experiments from the scatterplot by dragging a rectangle. The groups of experiments can be manipulated using various sets of operations: union, intersection, and difference. Previously defined manual annotations can be explored on the right side of the window using drop down options and several buttons. The website also provides an annotation browser where GEO annotations can be searched conveniently using mouse clicks.

## 4 DISCUSSION

Integrative data analysis platforms where all publicly available databases with diverse data types are coherently put together to propose novel hypotheses for ongoing deep investigation of biological processes is key for success in this new era of genomic data revolution. Tools that are developed in both plant community and human disease studies will significantly benefit each other. StepMiner (Sahoo et al., 2007), BooleanNet (Sahoo et al., 2008) and MiDReG (Sahoo et al., 2010) are examples of computational tools that were developed primarily to analyze human normal and cancer tissues are directly applicable in plant studies because the data characteristics are similar. Microarray data in human tissues and the plant tissues can be processed similarly. In this paper, we identify Boolean relationships between *Arabidopsis* genes; some of the genes are homologous to human genes. The comparison of data processing steps that we performed here will also benefit human studies.

Data processing steps strongly influence the downstream analysis. In this context, the choice of normalization steps has been debated before (Harr & Schlotterer, 2006; Lim, Wang, Lefebvre, & Califano, 2007). In this study, we conclude that RMA is more appropriate than MAS 5.0 for the investigation of Boolean logical gene‐gene relationships. ATH1 Affymetrix platform was extremely popular for initial transcriptomics studies within the Arabidopsis community. However, it contains a set of 22K probeset IDs, whereas the latest annotation of genes in this species is around 32K (Swarbreck et al., 2008). Therefore, it should be noted that the ATH1 platform may be missing well over 20% of Arabidopsis transcriptomic information. ATH1 experiments are being replaced by RNASeq alternatives nowadays. We show that RNASeq studies are also amenable for Boolean analysis by using log transformed TPM values.

A recent study focused on microarray and RNA‐seq based global and targeted co‐expression networks in *Arabidopsis* (Liesecke et al., 2018). This study identified Pathway Level Co‐expression using a set of guide genes, and compared how Pearson Correlation Coefficient (PCC), Spearman Correlation Coefficient (SCC), their respective ranked values (Highest Reciprocal Rank (HRR)), Mutual Information (MI) and Partial Correlations (PC) performed on global networks. In another study, a co‐expression database for plant species ATTED‐II (http://atted.jp) was published to aid in the discovery of relationships of unknown genes within a species (Obayashi, Aoki, Tadaka, Kagaya, & Kinoshita, 2018). ATTED‐II (version 9) provides 16 co‐expression platforms for nine plant species, including seven species supported by both microarray‐ and RNA sequencing (RNAseq)‐based co‐expression data (Obayashi et al., 2018). Similarly, co‐expression networks have been a popular tool in the literature to understand regulatory pathways in *Arabidopsis* (He & Maslov, 2016; Van Bel & Coppens, 2017; Zheng et al., 2017). All of the above studies focused on symmetric relationships between genes. However, our approach suggests that majority of the interesting biological information is present in the asymmetric relationships between genes which are often blurred in the co‐expression network investigations. Boolean relationships have been used to understand cell fate decisions in both normal (Inlay et al., 2009; Sahoo et al., 2010) and cancer tissues (Dalerba et al., 2011; Sahoo, 2012; Volkmer et al., 2012). Boolean relationships have been used to identify important biomarkers in colon cancer which was published in the New England Journal of Medicine (Dalerba, Sahoo, & Clarke, 2016; Dalerba, Sahoo, Paik, et al., 2016).

In summary, largescale global network analyses have tremendous potential in influencing the way plant biological investigations are approached. Co‐expression networks have been influential in this process. We sincerely believe that Boolean implication networks will benefit the ongoing investigations of plant biological processes by the plant community. We have provided several useful webservers and software packages to help biologists to systematically analyze their high‐throughput transcriptome data. We will constantly revise these software packages to make them more user‐friendly and effective based on users’ suggestions, comments, and recommendations.

## AUTHORS CONTRIBUTION

DS collected data and processed them for analysis. SP, DS analyzed data, made figures, and wrote manuscript.

## Data Availability

GEO Accession No: GSE118579. http://hegemon.ucsd.edu/plant.
