# Supplementary material for: Identification of gene expression logical invariants in Arabidopsis
Source: Plant Direct. 2019 Mar 20;3(3):e00123. doi: 10.1002/pld3.123 (PMC6508763; doi:10.1002/pld3.123)
Supplement: Supplementary file 1 [file PLD3-3-e00123-s001.pdf]

DuplicatedCEL	MD5-hash	CEL-file
./GSE26266/GSM645096.CEL.gz	76fb5e1716ca1333a52548c3207b7976	./GSE44655/GSM1088435_cc2.CEL.gz
./GSE26266/GSM645095.CEL.gz	b853018f7e1fe02ad174df9ed38125e8	./GSE44655/GSM1088442_ch1.CEL.gz
./GSE26266/GSM645101.CEL.gz	a6fb621ddc6e3ec5d3b7512c61ca7d02	./GSE44655/GSM1088438_qc1.CEL.gz
./GSE26266/GSM645093.CEL.gz	1ef134c969676d7ffdee4fce65c18520	./GSE44655/GSM1088434_cc1.CEL.gz
./GSE26266/GSM645103.CEL.gz	579039a672a51d97a992c55e13e1f9db	./GSE44655/GSM1088446_qh1.CEL.gz
./GSE26266/GSM645104.CEL.gz	8230a232ba248cc37c717658045094d9	./GSE44655/GSM1088447_qh2.CEL.gz
./GSE26266/GSM645094.CEL.gz	deb1b737d17dfd846c73d58ec1dea134	./GSE44655/GSM1088443_ch2.CEL.gz
./GSE26266/GSM645102.CEL.gz	5389f988eaca19c0cd81fe798bdd9e45	./GSE44655/GSM1088439_qc2.CEL.gz
./GSE21684/GSM540914.CEL.gz	d6ef20c09a8e1b82fa743918217d4c3f	./GSE6906/GSM159298.CEL.gz
./GSE21684/GSM540912.CEL.gz	7ff1cc970814a40239afd41d1762e3fe	./GSE6906/GSM159291.CEL.gz
./GSE21684/GSM540910.CEL.gz	1f38e9da341083faa136c96af63f3827	./GSE6906/GSM159273.CEL.gz
./GSE21684/GSM540922.CEL.gz	491cc4888b45cd6c86e8b95820dceabb	./GSE6906/GSM159302.CEL.gz
./GSE21684/GSM540924.CEL.gz	f78b86a771db753c0737706657038407	./GSE6906/GSM159304.CEL.gz
./GSE21684/GSM540926.CEL.gz	be0c7d0a4c2abb583ff3edde4d27679d	./GSE6906/GSM159321.CEL.gz
./GSE21684/GSM540908.CEL.gz	625e03a030602c1d87a92ea2d2512e57	./GSE6906/GSM158681.CEL.gz
./GSE21684/GSM540918.CEL.gz	30309bc0620479a879d35eaf23021872	./GSE6906/GSM159300.CEL.gz
./GSE21684/GSM540906.CEL.gz	09150b80ccb124f60b04608f5c34ce8f	./GSE6906/GSM158680.CEL.gz
./GSE21684/GSM540920.CEL.gz	390da52cfedebecb4f6f1cf64625c7a6	./GSE6906/GSM159301.CEL.gz
./GSE21684/GSM540916.CEL.gz	7d9de7aaaf171bdf51e311a18369036b	./GSE6906/GSM159299.CEL.gz
./GSE21684/GSM540928.CEL.gz	7191e7d41e2fc5c3157285aa01f640a3	./GSE6906/GSM159326.CEL.gz
./GSE6179/GSM142889.CEL.gz	ec70a102cf8159c448ac9b3d685a8cd7	./GSE6179/GSM142887.CEL.gz
./GSE39268/GSM959138_BrW_ATH1_121410_15_KpR24.CEL.gz	e38fde045161b2883a6f610adc0d7c0d	./GSE29086/GSM720635.CEL.gz
./GSE39268/GSM959127_BrW_ATH1_121410_01_TpR24.CEL.gz	cd06cede961a38f61fad1eca88fe4595	./GSE29086/GSM720624.CEL.gz
./GSE39268/GSM959143_BrW_ATH1_121410_23_KmR48.CEL.gz	218281ab9866c11909480a43eb8265cd	./GSE29086/GSM720640.CEL.gz
./GSE39268/GSM959142_BrW_ATH1_121410_22_KmR48.CEL.gz	caab27f9f95f6b7eda6d289176ef164e	./GSE29086/GSM720639.CEL.gz
./GSE39268/GSM959144_BrW_ATH1_121410_24_KmR48.CEL.gz	c824267d9d4a5f7bf64f0a3355088828	./GSE29086/GSM720641.CEL.gz
./GSE39268/GSM959133_BrW_ATH1_121410_10_TmR48.CEL.gz	ab0a97ed8f126dd75fcbf510d21c8f46	./GSE29086/GSM720630.CEL.gz
./GSE39268/GSM959129_BrW_ATH1_121410_03_TpR24.CEL.gz	44dc23c3ddc86e2a150d6713d842740f	./GSE29086/GSM720626.CEL.gz
./GSE39268/GSM959136_BrW_ATH1_121410_13_KpR24.CEL.gz	969c68524cd068e810cb3d5730b4ff1b	./GSE29086/GSM720633.CEL.gz
./GSE39268/GSM959137_BrW_ATH1_121410_14_KpR24.CEL.gz	8bc9b11cb534568763f50271c7762dfb	./GSE29086/GSM720634.CEL.gz
./GSE39268/GSM959132_BrW_ATH1_121410_06_TmR24.CEL.gz	9f6ff13496525a0bb6e4f4d6ded017d4	./GSE29086/GSM720629.CEL.gz
./GSE39268/GSM959141_BrW_ATH1_121410_18_KmR24.CEL.gz	96a5c18e503e362735968a2f8a6662cb	./GSE29086/GSM720638.CEL.gz
./GSE39268/GSM959131_BrW_ATH1_121410_05_TmR24.CEL.gz	c43f19fdd42ccc8afac3b169d3018ba1	./GSE29086/GSM720628.CEL.gz
./GSE39268/GSM959135_BrW_ATH1_121410_12_TmR48.CEL.gz	66bbfa1591a9fa95dc6e1d682104bdc0	./GSE29086/GSM720632.CEL.gz
./GSE39268/GSM959128_BrW_ATH1_121410_02_TpR24.CEL.gz	6fff24f6e82fc362549c726326578bbe	./GSE29086/GSM720625.CEL.gz
./GSE39268/GSM959140_BrW_ATH1_121410_17_KmR24.CEL.gz	176a731fd6ed93e8cdd370668364ef86	./GSE29086/GSM720637.CEL.gz
./GSE39268/GSM959130_BrW_ATH1_121410_04_TmR24.CEL.gz	a828ae45a01d0c31a0cee2c15dfbd115	./GSE29086/GSM720627.CEL.gz
./GSE39268/GSM959134_BrW_ATH1_121410_11_TmR48.CEL.gz	dedddc577f4ca08fcda0130f2766514d	./GSE29086/GSM720631.CEL.gz
./GSE39268/GSM959139_BrW_ATH1_121410_16_KmR24.CEL.gz	da57c834d0d3688f6e89b7fd0a8e32c0	./GSE29086/GSM720636.CEL.gz
./GSE627/GSM9572.cel.gz	de86cfb680522769b6d8636c0dbfd669	./GSE629/GSM9598.cel.gz
./GSE627/GSM9574.cel.gz	633608fc5043cba4a93da2c85acd4dc0	./GSE629/GSM9596.cel.gz
./GSE627/GSM9573.cel.gz	1df1629598c0cdd791e2854938a85a0a	./GSE629/GSM9597.cel.gz
./GSE627/GSM9576.cel.gz	bfc687cb394edf7be90377ed5db3f39e	./GSE629/GSM9600.cel.gz
./GSE627/GSM9575.cel.gz	ff40e0a744bcba6c36252f9a5d7d39f9	./GSE629/GSM9599.cel.gz
./GSE627/GSM9571.cel.gz	407983c94e3d6ea27c9d4940be65a431	./GSE629/GSM9595.cel.gz
./GSE31587/GSM784838_WT-D-3.CEL.gz	33843907b8949ebd2f51ec0ab987c85f	./GSE8951/GSM226268.CEL.gz
./GSE31587/GSM784841_WT-R50-2.CEL.gz	96c64327623823610e6e105ff89e2666	./GSE8951/GSM226279.CEL.gz
./GSE31587/GSM784840_WT-R50-1.CEL.gz	e2c6b9aa467c8f08f65d6127064173ab	./GSE8951/GSM226278.CEL.gz
./GSE31587/GSM784837_WT-D-2.CEL.gz	f60dc82b7b34d9720110364285644225	./GSE8951/GSM226267.CEL.gz
./GSE31587/GSM784839_WT-D-4.CEL.gz	b2ece6d69cc096e7a95b05c32dbdc061	./GSE8951/GSM226269.CEL.gz
./GSE631/GSM9624.cel.gz	de86cfb680522769b6d8636c0dbfd669	./GSE629/GSM9598.cel.gz
./GSE631/GSM9627.cel.gz	1df1629598c0cdd791e2854938a85a0a	./GSE629/GSM9597.cel.gz
./GSE631/GSM9625.cel.gz	633608fc5043cba4a93da2c85acd4dc0	./GSE629/GSM9596.cel.gz
./GSE631/GSM9626.cel.gz	ff40e0a744bcba6c36252f9a5d7d39f9	./GSE629/GSM9599.cel.gz
./GSE631/GSM9620.cel.gz	407983c94e3d6ea27c9d4940be65a431	./GSE629/GSM9595.cel.gz
./GSE631/GSM9628.cel.gz	bfc687cb394edf7be90377ed5db3f39e	./GSE629/GSM9600.cel.gz
./GSE34130/GSM842202.CEL.gz	7ba3b6688260b4136f979e560091a3c6	./GSE7631/GSM184539.CEL.gz
./GSE36789/GSM901073_10MR2_051209.CEL.gz	108e8e047e2e346eb25acd780210f172	./GSE33790/GSM835818.CEL.gz
./GSE36789/GSM901069_10ML1_051209.CEL.gz	0708c0d3be9de61c81c0018716aea485	./GSE33790/GSM835808.CEL.gz
./GSE36789/GSM901074_10MR3_051209.CEL.gz	4ec7115a288d89af357b2384b8115dcc	./GSE33790/GSM835819.CEL.gz
./GSE36789/GSM901072_10MR1_051209.CEL.gz	f15ccb781a8fc8d493c2936e2254800e	./GSE33790/GSM835817.CEL.gz
./GSE36789/GSM901071_10ML3_051209.CEL.gz	e2493eb2f8a7b7acbf626587995e96e1	./GSE33790/GSM835810.CEL.gz

./GSE14091/GSM353585.CEL.gz	cd3bd32725bd411ea249c3bbd2f9a75f	./GSE14091/GSM353590.CEL.gz
./GSE14091/GSM353589.CEL.gz	9448996c0b738246ba201ec98f6f0812	./GSE14091/GSM353584.CEL.gz
./GSE26983/GSM664663_Col_gl_-100mM-1.CEL.gz	bbb9326b6c117364daaa8bec7452fc32	./GSE16765/GSM420242_1910-RG.CEL.gz
./GSE26983/GSM664647_WS-100mM-1.CEL.gz	360312b50e7c924163ba02e0b80f274c	./GSE16765/GSM420235_2063-RG.CEL.gz
./GSE26983/GSM664644_Ws-0mM-2.CEL.gz	b7ae2356b73365ccab5d5d51f903eb38	./GSE16765/GSM420233_2096-RG.CEL.gz
./GSE26983/GSM664664_Col_gl_-100mM-2.CEL.gz	a5152b03e2a72250fe56eaeace16601f	./GSE16765/GSM420243_2097-RG.CEL.gz
./GSE26983/GSM664655_Col-100mM-1.CEL.gz	03ea3e1fb07b357257c4ab9b2b96dbf1	./GSE16765/GSM420238_1902-RG.CEL.gz
./GSE26983/GSM664656_Col-100mM-2.CEL.gz	293cfbcd9816cf673042bb9910d72533	./GSE16765/GSM420239_2064-RG.CEL.gz
./GSE26983/GSM664648_WS-100mM-2.CEL.gz	cce8379b6cf5d94556420d72e19a3544	./GSE16765/GSM420234_1906-RG.CEL.gz
./GSE26983/GSM664659_Col_gl_-0mM-1.CEL.gz	5fba2396e369f120ad0684d2eca381d4	./GSE16765/GSM420240_1880-RG.CEL.gz
./GSE26983/GSM664660_Col_gl_-0mM-2.CEL.gz	46f5dd5b41ba33c19043a9a0ad0a8dfb	./GSE16765/GSM420241_1908-RG.CEL.gz
./GSE26983/GSM664651_Col-0mM-1.CEL.gz	f2f1176ab01069f9a401f7b1963bd5cd	./GSE16765/GSM420236_1874-RG.CEL.gz
./GSE26983/GSM664643_WS-0mM-1.CEL.gz	46ca2cef660b23747dba75f0da8c260d	./GSE16765/GSM420232_1904-RG.CEL.gz
./GSE26983/GSM664652_Col-0mM-2.CEL.gz	fb2efc0c75acd69e789174884d494e95	./GSE16765/GSM420237_1900-RG.CEL.gz
./GSE15689/GSM392180.CEL.gz	4d0cae20801286564c8d6b5a240ad6f4	./GSE37014/GSM908542_Col-0-16C-A.CEL.gz
./GSE15689/GSM392687.CEL.gz	8cee8335a9c90ef7ed56c3eec6c2338e	./GSE37014/GSM908547_Col-0-23C-C.CEL.gz
./GSE15689/GSM392686.CEL.gz	1a1d194ed0d1bffa2add2c9df144fb14	./GSE37014/GSM908546_Col-0-23C-B.CEL.gz
./GSE15689/GSM392273.CEL.gz	2fe296c995263ff5f60f6f120e91d402	./GSE37014/GSM908544_Col-0-16C-C.CEL.gz
./GSE15689/GSM392685.CEL.gz	357d26227232d0c7af94646cece79a7a	./GSE37014/GSM908545_Col-0-23C-A.CEL.gz
./GSE15689/GSM392263.CEL.gz	1c37526327fe2054914c5bb88763c3cb	./GSE37014/GSM908543_Col-0-16C-B.CEL.gz
./GSE16557/GSM413936.CEL.gz	594bca4b922a55fe25b70dc616cdc010	./GSE16557/GSM413942.CEL.gz
./GSE17824/GSM444740.CEL.gz	147f2b435eb3527dd48de821bbed43b9	./GSE17824/GSM444748.CEL.gz
./GSE17824/GSM444746.CEL.gz	d7da7037e818ec3b87a8d35d78579313	./GSE17824/GSM444738.CEL.gz
./GSE17824/GSM444737.CEL.gz	7a75018af4bf5b6c8e64365cd6b9d89b	./GSE17824/GSM444745.CEL.gz
./GSE17824/GSM444739.CEL.gz	8f71ebcdb4a2c17d636ca7fe006673ce	./GSE17824/GSM444747.CEL.gz
