# Supplementary material for: Identification of gene expression logical invariants in Arabidopsis
Source: Plant Direct. 2019 Mar 20;3(3):e00123. doi: 10.1002/pld3.123 (PMC6508763; doi:10.1002/pld3.123)
Supplement: Supplementary file 2 [file PLD3-3-e00123-s002.pdf]

Title: Identification of gene expression logical invariants in *Arabidopsis*

Authors: Sonalisa Pandey[1], Debashis Sahoo[1,*]

Institutions: [1]University of California San Diego, CA, USA

Corresponding Author: [*]Debashis Sahoo, dsahoo@ucsd.edu

Keywords: Bioinformatics, microarray, Boolean analysis, Systems Biology

Response to Reviewers:

We appreciate the positive and enthusiastic feedback from the reviewers. Following are our detailed point-by-point response to the reviewers' comments in italics.

-- Reviewer comments:

Reviewer #1:

Authors present a platform for the analysis of pairwise co-expression patterns for Arabidopsis genes, based on a set of Affymetrix ATH1 expression datasets. Authors present a thorough collection and analysis of .CEL files existing datasets, and infer on proper gene normalization methods. Overall, the strategy seems sound and well executed. The innovative nature of this work regards the implementation of Boolean logic to identify gene co-expression patterns. This is in clear contrast with the numerous previous efforts within the plant community, which address gene co-expression on a global or targeted way, by usually applying correlation coefficients or mutual ranking mathematical approaches. These will provide a more linear response for the detection of co- or anti-expression, whereas Boolean logic has the potential to pinpoint more subtle co-expression patterns that may still provide significant biological insight. Authors, who have an extensive track record in human biology, provide a web-based resource that is novel and useful to the plant (and more specifically the Arabidopsis) functional biology community. The MS is well written and suited for publication. However, there are a few minor issues that I believe should be addressed prior to publication, that regard the contextualization of the approach within the present set of plant co-expression resources.

We really appreciate the time and effort put by the reviewers to review our manuscript and write a detailed long review. In the revised manuscript we address all of the concerns raised.

1. Authors use the Affymetrix ATH1 microarray datasets. As such they must acknowledge the inherent limitation that this microarray provides: ATH1 was the universal platform for initial transcriptomics studies within the Arabidopsis community, but it contains a set of 22K probes, whereas the latest annotation of genes in this species is circa 29K. Hence, their present platform may be missing well over 20% of Arabidopsis transcriptomic information. This must be mentioned in the MS.

We have added this in the revised discussion section.

2. The community has abandoned ATH1 for RNA-Seq approaches for at least 5 y now. How do authors plan to develop their resource to gear it towards the incorporation of the growing body of RNA-Seq data? Resources such as ATTED-II already have co-expression data based on RNA-Seq data, and other resouces such as BAR/Virtual Plant already incorporate gene expression atlas from RNA-Seq data.

We have revised Figure 3 by adding a new RNASeq dataset. We show that Boolean analysis can also be performed in RNASeq dataset and show consistent Boolean implication relationships compared to microarray dataset. We have revised our methods, results and discussion section to incorporate the reviewer's comment.

3. In line with this, and analyzing the Methods section for the collection of datasets, then the claim in the Abstract and Significance sections for the "incorporation of all publicly available Arabidopsis datasets" is too strong, and must be toned down.

We thank the reviewer to point out this mistake. We have corrected the statements.

4. The Genemania App in Cytoscape also incorporates co-expression datasets, and could be incorporated into the MS's literature overview.

We have added this in the revised introduction section.

4. As innovative as the mathematics are, within the context of present plant/Arabidopsis co-expression resources, it is not as integrative and interlinked as other databases. A major issue is the fact that, from the beginning of the genome sequencing in 2000, the Arabidopsis community and the numerous ensuing databases have been centered on the precision implicated in the Arabidopsis Gene ID code (AGI code, AT#G#####). The AGI code is the universal query term for almost all Arabidopsis databases. Yet the present resource is centered around the ATH1 probe_ID or the gene name. This is a limitation that should be addressed by authors in a new iteration of the resource, plus it opens up the possibility of automatically linking genes to other databses (otherwise they mail fail to get the expected attention from the Arabidopsis community, that their mathematical approach merits). Perhaps this issue should also be mentioned in the present MS, when authors present their new web-based resource.

We have added this feature in our web resource. Now it accepts ATH1 probeset ID, gene symbol name and AGI code to show scatterplots.

Reviewer #2:

The manuscript submitted by Pandey et al represents an important tool for the Arabidopsis community.

This work applies the boolean implication networks to a large set of public microarray experiments with the ability to identify asymmetric gene expression relationship. This method has been applied successfully to identify disease markers in animals.

We thank the reviewer for the enthusiastic response.

Although the method and the statistical approach described in this manuscript has been published a decade ago by Sashoo et al, the current manuscript lacks of important details that will allow the readers to replicate the results.

We have added github links, GEO accession number for the reviewer to replicate the results.

In particular, the work does not explain how the array datasets are handled- how the biological replicates have been considered in the study; what kind of genes were considered (protein-coding genes or noncoding -genes), how the statistical tools were applied. What kind of scripting language has been used to make the network.

We have tried to remove duplicate entries from the dataset. However, biological replicates are handled just like an independent sample. For the microarrays, we considered all probesets that have a good dynamic range of gene expression values. For the RNASeq data we use the gene annotation file provided to compute the TPM values. Both the microarray as well as the RNASeq dataset include protein coding as well as non-coding genes. All the software resources including github links are posted in the web resource.

The authors also provided in the manuscript a web tool to interrogate the Arabidopsis data but no clear instructions have been provided to help the reader to test the tool and to get clue about the data.

We have added a tutorial section in the web resource that will help the reader to test the tool.
